# Assay to Detect Oseltamivir Resistance

**DOI:** 10.3201/eid1212.060673

**Published:** 2006-12

**Authors:** Kamol Suwannakarn, Salin Chutinimitkul, Sunchai Payungporn, Thaweesak Chieochansin, Apiradee Theamboonlers, Alongkorn Amonsin, Sudarat Damrongwatanapokin, Le Quynh Mai, Nguyen Hong Hanh, Yong Poovorawan

**Affiliations:** * Chulalongkorn University, Bangkok, Thailand;; † National Institute of Animal Health, Bangkok;; ‡ National Institute of Hygiene and Epidemiology, Hanoi, Vietnam

**Keywords:** Oseltamivir, H5N1, influenza A, avian influenza, detection, resistance, letter

To the Editor: Oseltamivir is a neuraminidase inhibitor approved for treatment and prevention of influenza virus infection. Oseltamivir resistance caused by a single amino acid substitution from histidine (H) to tyrosine (Y) at position 274 of the neuraminidase active site has been reported in persons infected both experimentally and naturally with influenza A virus subtype H5N1 (*1**,**2*). Evidence suggests that using lower doses of oseltamivir or shorter-than-recommended treatment periods can contribute to emergence of viral resistance (*1**,**3*) Currently, oseltamivir is being used in several countries that may be affected by epidemics of H5N1. Therefore, monitoring for oseltamivir-resistant strains of H5N1 during oseltamivir administration is essential for outbreak management and prevention.

Although real-time PCR or pyrosequencing is more rapid than high-throughput assays for mutation detection (*4**,**5*), the conventional PCR technique can be applied to detect drug-resistant mutation (*6*) in areas lacking real-time PCR or pyrosequencing capabilities. Therefore, to discriminate between oseltamivir-sensitive and oseltamivir-resistant strains, we developed a simple method, based on PCR, which takes advantage of the H274Y substitution. The forward primer was designed from the conserved region common to both wild-type and mutant strains; the reverse primers were designed specifically for wild-type and mutant strains, respectively, derived from the 3´ terminal base of each primer. The primers consisted of a forward primer N1f (nt 517-534: 5´-GGGGCTGTGGCTGTATTG-3´) and reverse primer H274r (nt 759-784: 5´-GGATAACAGGAGCAYTCCTCATAGTG -3´) for wild-type strain detection or Y274r (nt 759-784: 5´-GGATAACAGGAGCAYTCCTCATAGTA-3´) for mutant strain detection. Both strains yielded products of ≈267 bp; hence, the assay consisted of 2 separate reactions for detecting wild-type and mutant strains, respectively.

For each reaction, 1.0 μL cDNA was combined with a reaction mixture that contained 10 μL 2.5× MasterMix (Eppendorf, Hamburg, Germany), forward and reverse primers at a final concentration of 0.15 μM, and nuclease-free water to a final volume of 20 μL. Thermocycling conditions comprised initial denaturation at 94°C for 3 min and 35 cycles of amplification including denaturation (94°C, 30 s), annealing (65°C, 50 s), extension (72°C, 45 s), and final extension (72°C, 7 min). Subsequently, 10 μL of the amplified products was analyzed by using 2% agarose gel electrophoresis.

To optimize the assay, we performed PCR-based H274Y mutagenesis of the N1 fragment of the H5N1 virus (primers on request). The resulting mutagenic and wild-type products were cloned into the pGEM-T Easy Vectors (Promega, Madison, WI, USA), confirmed by direct sequencing, and then used as positive controls. Preliminary results showed that the wild-type primer was specific for the oseltamivir-sensitive strain, whereas the mutant primer can be used to detect the oseltamivir-resistant strain exclusively because no significant cross-amplification had been observed.

To establish sensitivity, serial 10-fold dilutions of the standard N1 plasmids (wild-type and mutant) ranging from 10^9^ to 10^1^ copies/μL were used as a template. The threshold concentrations for detection of wild-types and mutants were 10^3^ copies/μL. To provide semiquantitative data to detect subpopulations of the resistant variants, the 2 control plasmids were mixed at wild-type:variant ratios of 10^8^:10^2^, 10^7^:10^3^, 10^6^:10^4^, 10^5^:10^5^, 10^4^:10^6^, 10^3^:10^7^, and 10^2^:10^8^. The result showed that the density of the expected bands depended on the amount of DNA templates (Figure, B). However, the mixing experiments indicated that the predominant mixtures of wild-type:resistant variant were 80:20, which is the lowest ratio of resistant variants that the assay can reliably detect (data not shown). To assess specificity, human DNA and viral cDNA extracted from other subtypes of influenza A virus (N2–N9) were subjected to this assay. No cross-reaction occurred with human DNA or other subtypes of influenza A virus.

**Figure:**
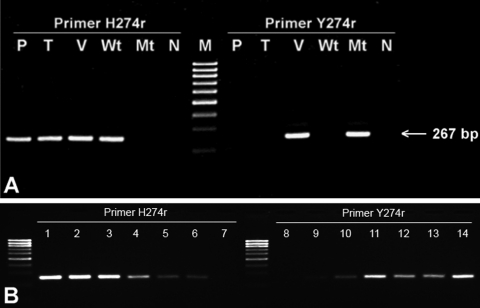
A) Representative result from conventional PCR that used H274r primer for oseltamivir-sensitive and Y274r primer for oseltamivir-resistant detection in samples isolated from human plasma (P), tiger (T), and Vietnamese patient (V). Plasmids containing N1 fragments obtained from PCR-based mutagenesis for wild-type H274 (Wt) and mutant Y274 (Mt) were used as positive controls in each reaction. (N, no template control; M, 100-bp molecular marker.) B) Semiquantitative data on the ability of the assay to detect subpopulations of the resistant variants. The 2 control plasmids were mixed at wild-type:variant ratios of 10^8^:10^2^ (lanes 1 and 8), 10^7^:10^3^ (lanes 2 and 9), 10^6^:10^4^ (lanes 3 and 10), 10^5^:10^5^ (lanes 4 and 11), 10^4^:10^6^ (lanes 5 and 12), 10^3^:10^7^ (lanes 6 and 13), and 10^2^:10^8^ (lanes 7 and 14).

We further validated the assay by testing 3 specimens from hosts treated with oseltamivir and 17 specimens from untreated hosts; infection with H5N1 was detected by using multiplex real-time PCR (*7*). The specimens from oseltamivir recipients were isolated from a Vietnamese patient on the third day after he received a prophylactic dose (*1*) and from 2 tigers (CU-T7 and KU-11) (*8*). The specimens from untreated hosts were isolated from the plasma of an H5N1-infected human (*9*) and from virus-containing allantoic fluid isolated from infected chickens, ducks, and cats (n = 16) during a 2005 outbreak in Thailand. The specimen isolated from the Vietnamese patient yielded detectable bands after amplification by wild-type and mutant primer sets, which indicates that this specimen contains mixed populations of wild-type and resistant strains (Figure, A). The result was confirmed by cloning the amplicon of the Vietnamese strain into the pGEM-T EASY vector (Promega). Ten colonies were randomly picked and sequenced; 9 clones were mutant, and 1 was wild type. The other specimens produced a visible positive band only on amplification using the wild-type primer set, which indicates that samples containing these strains were oseltamivir sensitive.

The assay described here provides an accurate, cost-effective, and highly efficient approach to detecting oseltamivir-sensitive and oseltamivir-resistant H5N1 strains; it is based on conventional PCR and takes advantage of the H274Y substitution within the neuraminidase gene. This simple technique can be applied in all laboratories that lack more advanced and expensive instruments; thus, it provides a valuable tool for managing and preventing influenza A H5N1 outbreaks. Concerning the spread of mutant viruses, however, the fitness of the viruses needs further investigation.
